# An mHealth Intervention to Reduce the Packing of Discretionary Foods in Children’s Lunch Boxes in Early Childhood Education and Care Services: Cluster Randomized Controlled Trial

**DOI:** 10.2196/27760

**Published:** 2022-03-17

**Authors:** Nicole Pearson, Meghan Finch, Rachel Sutherland, Melanie Kingsland, Luke Wolfenden, Taya Wedesweiler, Vanessa Herrmann, Sze Lin Yoong

**Affiliations:** 1 Hunter New England Population Health Wallsend Australia; 2 School of Medicine and Public Health University of Newcastle Callaghan Australia; 3 Hunter Medical Research Institute Newcastle Australia; 4 Priority Research Centre for Health Behaviour University of Newcastle Callaghan Australia

**Keywords:** nutrition, mHealth, child, preschool, parents

## Abstract

**Background:**

Interventions in early childhood education and care (ECEC) services have the potential to improve children’s diet at the population level.

**Objective:**

This study aims to test the efficacy of a mobile health intervention in ECEC services to reduce parent packing of foods high in saturated fat, sugar, and sodium (discretionary foods) in children’s (aged 3-6 years) lunch boxes.

**Methods:**

A cluster randomized controlled trial was undertaken with 355 parent and child dyads recruited by phone and in person from 17 ECEC services (8 [47%] intervention and 9 [53%] control services). Parents in the intervention group received a 10-week fully automated program targeting barriers to packing healthy lunch boxes delivered via an existing service communication app. The program included weekly push notifications, within-app messages, and links to further resources, including websites and videos. The control group did not receive any intervention. The primary outcomes were kilojoules from discretionary foods and associated nutrients (saturated fat, free sugars, and sodium) packed in children’s lunch boxes. Secondary outcomes included consumption of kilojoules from discretionary foods and related nutrients and the packing and consumption of serves of discretionary foods and core food groups. Photography and weights of foods in children’s lunch boxes were recorded by trained researchers before and after the trial to assess primary and secondary outcomes. Outcome assessors were blinded to service allocation. Feasibility, appropriateness, and acceptability were assessed via an ECEC service manager survey and a parent web-based survey. Use of the app was assessed via app analytics.

**Results:**

Data on packed lunch box contents were collected for 88.8% (355/400) of consenting children at baseline and 84.3% (337/400) of children after the intervention. There was no significant difference between groups in kilojoule from discretionary foods packed (77.84 kJ, 95% CI −163.49 to 319.18; *P*=.53) or the other primary or secondary outcomes. The per-protocol analysis, including only data from children of parents who downloaded the app, also did not find any statistically significant change in primary (−1.98 kJ, 95% CI −343.87 to 339.90; *P*=.86) or secondary outcomes. Approximately 61.8% (102/165) of parents in the intervention group downloaded the app, and the mean service viewing rate of weekly within-app messages was 26% (SD 14.9). Parents who responded to the survey and participating services agreed that it was appropriate to receive lunch box information via the app (40/50, 80% and 6/8, 75%, respectively).

**Conclusions:**

The intervention was unable to demonstrate an impact on kilojoules or associated nutrients from discretionary foods packed in children’s lunch boxes. Low app downloads and program message views indicate a need to explore how to improve factors related to implementation before further testing similar mobile health interventions in this setting.

**Trial Registration:**

Australian New Zealand Clinical Trials Registry ACTRN12618000133235; https://www.anzctr.org.au/Trial/Registration/TrialReview.aspx?id=374379

## Introduction

### Background

Poor diet characterized by excessive intake of foods high in sugar, salt, and sodium and low intake of core foods such as fruits and vegetables is associated with a higher risk of chronic disease [1]. The consumption of foods high in saturated fat, sugar, or salt exceeds optimal levels worldwide [2], and in countries such as the United States, the United Kingdom, and Australia, excessive consumption of such nutrients has been reported across all age groups [3-5]. In Australia, foods high in saturated fat, sugar, or sodium, and low in nutrients such as fiber (from here on referred to as *discretionary foods*) [6], contribute up to 38% of the total daily energy intake of a child aged 4 to 8 years [5]. Given this, international and national dietary guidelines recommend limiting the consumption of discretionary foods [7-10].

Early childhood education and care (ECEC) services constitute an ideal setting for reaching and engaging families to influence lifelong eating habits [11,12]. In the United States, the United Kingdom, and Australia, up to 80% of children attend some kind of formal ECEC service in the year before school [13-15], with an average attendance of 2 to 3 days per week [15]. A significant number of ECEC services in these countries require children to bring food from home in a lunch box (approximately 30%-50%) [13,14,16]. Australian studies report that between 50% and 60% of lunch boxes include discretionary foods [17,18], with one reporting an average of 2 serves included per lunch box [18]. As such, there is potential to improve children’s diet by targeting the packing of discretionary foods in lunch boxes.

A systematic review of interventions to improve the contents of lunch boxes identified just 3 randomized controlled trials that examined interventions to improve the packing of discretionary foods within the ECEC setting [17,19,20]. Of the 3 trials, 2 reported significant results for reducing the packing of discretionary foods [17,19]. Both were intensive multicomponent interventions, including teacher education, child curriculum, and face-to-face education sessions with parents [17,19]. Despite the success of these interventions, evidence suggests that the sustainability and scalability of such intensive designs may be limited [21,22]. In particular, face-to-face parent-related components have been identified as difficult for parents to attend because of time and travel burden [23] and challenging for ECEC services to implement [21].

The use of digital health–delivered interventions has been proposed as a way of overcoming barriers to engaging parents in nutrition interventions, as well as enhancing scalability [24-26]. Digital health interventions (DHIs), in particular the use of mobile phone apps or mobile health (mHealth) interventions, have been identified as an effective way of providing health-related information to parents [27] and influencing child health outcomes [25,28-30]. In addition, mobile apps designed to facilitate parent ECEC service communications are increasingly available for use by ECEC services in Australia, offering an alternative to face-to-face delivery of nutrition interventions by drawing on existing digital platforms [26]. Using existing apps to deliver nutrition interventions has also been recommended to capitalize on technologies developed by experts in user-centered design and with established commercial appeal [26]. Therefore, integrating a lunch box intervention into an existing ECEC parent communication app shows promise as a novel way of delivering digital content and engaging with parents.

### Objective

To the authors’ knowledge, no trials have been conducted to assess whether mHealth interventions can support the packing of healthy lunch boxes for children attending ECEC services. To address this gap in research evidence, the aim of this study is to determine the efficacy of using an mHealth intervention versus no intervention in ECEC services to reduce the packing of discretionary foods in children’s lunch boxes. We hypothesize that as a result of the intervention, lunch boxes of children with parents in the intervention group would achieve a mean reduction of 123 kJ from packed discretionary foods relative to the control. Additional outcomes include the intervention’s impact on children’s consumption of food, parental use of the app, intervention fidelity, and other process evaluation measures.

## Methods

### Ethics Approval

Ethical approval was obtained from the Hunter New England (HNE) human research ethics committee (06/07/26/4.04) and ratified by the University of Newcastle ethics committee (H-2008-0343). The trial is reported in line with the CONSORT-EHEALTH (Consolidated Standards of Reporting Trials of Electronic and Mobile Health Applications and online Telehealth) reporting guidelines (Multimedia Appendix 1) [31], and a protocol has been previously published [32].

### Study Design and Setting

The study used a cluster randomized controlled trial design and targeted the parents of children attending center-based ECEC services (ie, long day care and preschools). The ECEC services were located in the HNE Local Health District of New South Wales (NSW), Australia. ECEC services were randomized to a 10-week intervention group or no intervention control group. Within NSW, long daycare services can provide center-based education and care for children aged from 6 weeks to 6 years for ≥8 hours per day. Preschools typically enroll children aged between 3 and 6 years and provide care for 6 to 8 hours per day [33]. In 2016, approximately 920,370 people were reported to live in the HNE area, with 51,900 of these being children aged 0 to 4 years [34]. The area includes major metropolitan centers and inner regional communities, with a small proportion (14%) of people in remote communities [35].

### Sample

The sampling frame comprised ECEC services from the region that required parents to provide food for consumption at the service (ie, lunch box services). The list of ECEC services that required parents to provide food was obtained from a government database and represented approximately 53% of all ECEC services in the region [16]. Of these services, existing users of the app required for the intervention and those not using any app (ie, services able to commence using the intervention app) were identified using previous, unpublished data on app use by services in the region.

### Recruitment and Eligibility

Recruitment for the trial occurred in 2 phases. Initially, ECEC services were eligible to participate in the trial if they enrolled children aged 3 to 6 years and were existing users of the designated parent communication app. As this eligibility criterion did not result in adequate ECEC services being recruited, phase 2 recruitment extended the eligibility criteria to include ECEC services not yet using the app but willing to commence using the app for the trial. For both phases, recruitment involved research assistants posting and emailing information statements and consent forms to ECEC services outlining the study, data collection procedures, and requesting participation. Written consent was obtained from service managers. Parents or carers (hereafter referred to as *parents*) of children aged 3 to 6 years were eligible to participate if their child attended during the designated days of data collection and if they used or indicated a willingness to download the intervention app on the consent form. To obtain parental consent for participation in the study, ECEC service staff distributed hard copies of parent information statements and consent forms. Parents could also consent on the day of the data collection.

### Random Allocation and Blinding

Before baseline data collection, ECEC services were randomly allocated to the intervention group or no intervention control group in a 1:1 ratio by a statistician independent of the trial using a computerized random number generator. Before randomization, ECEC services were stratified by rural location and socioeconomic status (SES) of the service, as evidence indicates that these factors are associated with family dietary intake [36,37]. To ensure equity of access to the intervention, ECEC services were also stratified by those with high numbers of Aboriginal child enrollments (defined as those with >10% Aboriginal children enrolled). Stratification by this number of factors was deemed appropriate for the sample size [38]. Owing to the nature of the intervention, ECEC services and parents were not blinded to the intervention; however, outcome assessors were blinded to the service allocation.

### Intervention Development

Details of the intervention and its development, including the application of theory, cultural, and other stakeholder consultation processes, have been published elsewhere [32]. Briefly, the intervention was based on an mHealth lunch box intervention originally designed for primary (elementary) schools by the research team, which reported promising pilot data on the packing of discretionary items in school [39,40]. Similar to the original intervention, the behavior change wheel (BCW) and COM-B (Capability, Opportunity, Motivation, and Behavior) model were used to inform the current intervention [41]. The BCW framework is based on 19 theories of health behavior change and facilitates the systematic development of behavior change interventions. The COM-B model is the behavior system behind the BCW framework, which supports the identification of essential conditions for changes, including capability, opportunity, and motivation [41]. The resulting mHealth intervention, *SWAP IT for Childcare*, comprised 7 behavior change techniques targeting 8 barriers to packing healthy lunch boxes. (Multimedia Appendix 2). Barriers were identified from the literature and formative interviews with a convenience sample of parents from 3 local ECEC services. The adaptation process was then guided by the Framework for Reporting Adaptations and Modifications–Enhanced (Multimedia Appendix 3) [42].

### Intervention App

The app used for the intervention (Skoolbag) was an existing app designed to be available for use by schools or ECEC services to communicate with parents. Commonly, ECEC services use the app to share parent newsletters, reminders, and other service information. The app had not been previously used to deliver nutrition interventions in the ECEC setting.

### Intervention Strategies

The *SWAP IT for Childcare* intervention comprised 3 components.

#### Provision of Weekly Push Notifications and Within-App Messages

A total of 11 push notifications messages were delivered over 10 weeks (1 per week, with an additional introductory message delivered in week 1). The push notifications were sent by the app provider. Each push notification alerted users to a within-app message, which aimed to target parent barriers to packing healthy lunch boxes. The message was accessible via the push notification or could be accessed as part of the static content within the app. A summary of each push notification, within-app message, and the behavior change techniques is available in the published protocol [32].

#### Provision of SWAP IT Options and Supporting Resources Via the App

Several of the within-app messages provided a weblink to *SWAP IT Options*—a comprehensive list of foods suitable for packing in the lunch box. The web-based list was developed by dietitians on the research team with expertise working within the setting. The lists were divided into sections (sweet snacks, savory snacks, and lunch foods) with drop-down tabs to explore foods recommended as *swap from* and *swap to*. Links to other supporting information relevant to each message were also provided, including fact sheets, short videos, and website links.

#### ECEC Service Endorsement of Program

To encourage initial and ongoing engagement with the app content, intervention ECEC service managers were asked to endorse the *SWAP IT* program and demonstrate support to parents. To do this, the research team provided 2 templates to service managers to communicate with parents during the course of the trial, which service managers could deliver via the app or by email. ECEC services were asked to deliver the communications the week before the intervention and midintervention (weeks 5-6).

#### Service Implementation Strategies to Increase App Uptake and Engagement

Although not an implementation trial, to maximize uptake and engagement with the intervention, some implementation strategies were undertaken [43].

##### Use Financial Strategies and Train Stakeholders

Before the intervention, ECEC services that had not used the app before the trial (intervention: 6/17, 35%; control: 5/17, 29%) had their access paid for by the research team during the trial period. Remote training was provided to new ECEC services on how to use the app by the app provider.

##### Engage Consumers

On recruitment, parents were supported to gain access to the app if not already doing so. Multiple strategies were used to maximize the number of parents on the app before and during the intervention. This included the research team providing emails to service managers to send to parents, as well as researchers sending emails sent directly to consenting parents asking them to download the app. Emails included step-by-step instructions on how to download the app and were distributed via printed flyers to ECEC services.

### Control

The ECEC services allocated to the control group received no intervention. ECEC services had access to the app to use for their own communications but did not receive any of the intervention strategies or content.

### Sample Size and Power Calculations

This study aimed to recruit 390 children from 18 ECEC services. Allowing a 15% attrition rate, this would enable the detection of a mean difference of 123 (SD 200) kJ in the primary outcome (kilojoules from discretionary foods), with an *α* of .01 (adjusting for multiple outcomes using Bonferroni adjustments) and an estimated intraclass correlation coefficient of 0.1 with 80% power. Such an energy reduction would represent approximately a quarter of a serve reduction in packed discretionary foods and could be expected to result in the detection of approximately 2.2 g less sugar, 0.6 g less saturated fat, and 44 mg less sodium [32].

### Measures and Data Collection Procedures

#### Overview

Service baseline data were collected between May 2018 and July 2018. The 10-week intervention was delivered between July and September 2018. Postintervention data were collected for child lunch box outcomes during October and November 2018, and parent data were collected from October to December 2018.

The primary outcome measures were mean energy (kJ) provided by discretionary foods and mean energy (kJ), saturated fat (g), free sugars (mg), and sodium (mg) provided by all foods packed in the lunch box. Secondary outcomes were mean energy from discretionary foods (kJ) and mean energy (kJ), saturated fat (g), free sugars (mg), and sodium (mg) from all foods consumed by children from their lunch box. Other secondary outcomes were the number of serves of core food groups (bread and cereals, fruits, vegetables, dairy, meat, and meat alternatives) and number of discretionary food serves packed and consumed by children.

#### Packing and Consumption of Foods from Lunch Boxes

To assess the effectiveness of the intervention, data on food contents packed and consumed from lunch boxes were collected by trained research assistants, on one day, via weighed food records before the child’s first meal and at the end of the child’s last meal. ECEC services were asked not to inform parents of the day of data collection to reduce performance bias on lunch box packing behaviors. Weighed food records were used as evidence indicates this is the most accurate way of capturing portion sizes and quantities eaten [44,45]. On the day of data collection, the children were instructed to leave all uneaten food in the lunch box. Lunch box contents were also recorded on written standardized forms and digitally photographed to verify weighed records and identify inedible waste. Food record data were extracted by trained dietitians and entered into a nutrition analysis database (FoodWorks) [46]. Consumption data were derived by subtracting the weight of the food left at the completion of the last meal from the weight of food packed before meals. FoodWorks was used to produce nutrient data and the core food group serves data for both packed and consumed amounts of food. The software uses core food group classifications and serve sizes consistent with the Australian Guide to Healthy Eating [6]. For foods that were homemade, an appropriate standard recipe was sourced from within the FoodWorks database. As FoodWorks does not have the function to calculate discretionary food serves, this was undertaken manually by trained dietitians, using definitions based on the Australian Guide to Healthy Eating [6]. If further guidance was required, discretionary food classifications were sourced from the National Nutrition Survey food classification databases [47] or consensus from the research team. Further details of the FoodWorks data extraction processes and analysis are reported elsewhere [48].

#### Parent and Service Characteristics

Parent-child dyad characteristics were collected via consent forms and as part of a wider web-based survey sent via parent email before the intervention. Service operational characteristics were collected via service manager pen and paper surveys completed at baseline. During a site visit, the service nutrition context was measured by trained observers using a modified version of the Environment and Policy Assessment Observation (EPAO) tool. The tool was adapted to include only nutrition items from the original EPAO tool relevant to ECEC services where foods are brought from home (ie, items related to services that provide food were omitted). Additional items were added related to staff monitoring of lunch boxes for discretionary foods, staff actions taken as a result of discretionary foods in lunch boxes, and the presence of specific lunch box guidelines at the service [49].

#### Process Evaluation

Intervention fidelity was captured via app analytics and through a service manager, self-completed written record. App analytics were used to access data on app downloads and the *SWAP IT* program use. Parent and service measures of acceptability and feasibility of the *SWAP IT* program were undertaken using a web-based survey and service manager pen and paper survey. Acceptability and feasibility questions were modified versions of items taken from the Acceptability of Intervention Measure and the Feasibility of Intervention Measure [50]. Questions to determine if any cointerventions occurred during the trial and capture any adverse events as a result of the intervention were included in the service manager pen and paper survey. Further details of the process data collection methods and items used within the surveys are provided in Multimedia Appendix 4 [50].

### Statistical Analysis

A statistician independent of the study performed all analyses using SAS (version 9.4) statistical software. Descriptive statistics were generated for service, parent, and child characteristics. The Australian Statistical Geography Standard Remoteness Structure [51] was used to classify parent residences as a major city, inner regional city, or outer city location. This classification was included as health disparities, including nutrition-related risk factors, exist between major city and inner regional cities (characterized by shorter distances by road to access services) and outer city locations (characterized by longer distances by road to services) [52].

A measure of between-group differences in primary and secondary outcomes was assessed for paired data using 2-level hierarchical linear regression models. Models were adjusted for potential ECEC service level clustering through a service random effect and controlled for baseline service EPAO score and if ECEC services were existing app users before the trial. An intention-to-treat analysis was undertaken [53], with multiple imputations undertaken for primary and secondary outcomes using the SAS MI and MIANALYZE procedure. Complete case analysis and prespecified subgroup analyses by child gender and SES were undertaken by adding a group by subgroup interaction fixed effect. SES was determined by parent postcodes and classified as being in the top or bottom 50% of NSW according to the Socioeconomic Indexes for Areas [54]. A sensitivity outcome analysis, modeled similarly to the complete case analysis, was undertaken whereby the effects of the intervention were assessed in parents known to have only downloaded the app (181/400, 51%). In addition to the analysis prespecified as part of the trial protocol [32], an exploratory analysis was performed to explore any association between how often the service routinely used the app (mean hours per week) and the parent viewing rate of within-app messages using Spearman rank order correlation.

## Results

### Sample

**Figure 1:**
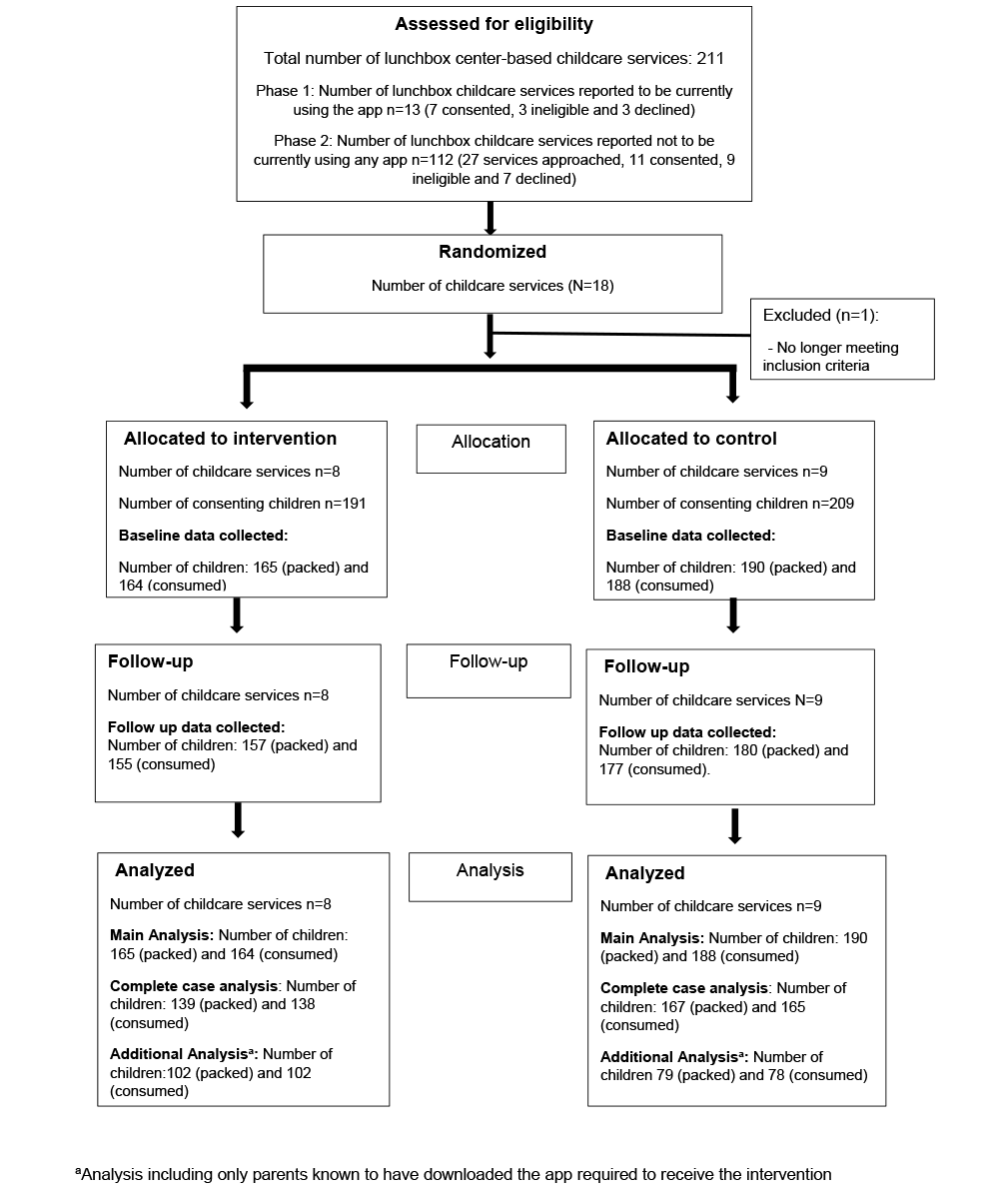
CONSORT (Consolidated Standards of Reporting Trials) flow diagram. ECEC: early childhood education and care.

The combined consent rate of ECEC services was 45% (18/40; 40 services approached to reach the required 18 services). A service in the intervention arm ceased using the required app and withdrew from the study (before baseline data but after random allocation). Within the 17 consenting ECEC services, 400 parent-child dyads consented to participate in the baseline data collection. The mean parent consent rate was 51%. Data on packed lunch box contents were collected for 88.8% (355/400) of the children at baseline and for 84.3% (337/400) after the intervention. Consumption data were collected for 88% (352/400) of children at baseline and for 84.3% (337/400) after the intervention (Figure 1). The number of parents from the intervention group completing the web-based survey after the intervention was 21.6% (41/190). Parents who completed the survey were less likely to have a Technical and Further Education certificate or diploma (*P*=.04) or be from an inner regional city. (*P*=.04). Postintervention service pen and paper surveys were completed by all intervention ECEC services.

### Parent and Service Characteristics

Baseline characteristics of ECEC services, parents, and children with lunch box data at baseline are reported in Table 1. Characteristics were similar across groups; however, the intervention group had a higher proportion of parents located in the outer regional areas than the control group. As the intervention was delivered at the cluster level rather than at the individual level, geographical remoteness was accounted for by stratifying services by this factor during randomization and not controlled for as part of the analysis. Before the intervention, 12% (2/17) of ECEC services in the intervention group were existing users of the app, and 35% (6/17) of ECEC services had not been using any app.

**Table 1:** Characteristics of ECEC^a^ services (n=17) and children (n=355).

Characteristics			Intervention		Control
**Service**					
	ECEC services, n (%)		8 (47)		9 (53)
	**ECEC service type**				
		Preschool	7 (88)	7 (78)	
		Long day care	1 (12)	2 (22)	
	Hours opened per day, mean (SD)		8.2 (1.54)		8.2 (0.88)
	Number of child enrollments, mean (SD)		68.1 (27.4)		68.7 (21.58)
	Modified EPAO^b^ score (score out of 20), mean (SD)^c^		18.35 (2.33)		16.63 (2.86)
**Parent and children dyads**					
	Dyads, n (%)		165 (46.5)		190 (53.5)
	Child age (years)^d^, mean (SD)		3.9 (0.68)		3.9 (0.67)
	**Child gender, n (%)**				
		Female	86 (52.1)	98 (51.6)	
		Male	79 (47.9)	92 (48.4)	
	**Parent SEIFA^e^ (based on postcode)^f^, n (%)**				
		Most disadvantaged	98 (62.8)	102 (55.7)	
		Least disadvantaged	58 (37.2)	81 (44.3)	
	**Parent education level^g^, n (%)**				
		Attended or completed high school	37 (23.4)	40 (22.2)	
		Technical or Further Education (TAFE) certificate or diploma	50 (31.6)	59 (32.8)	
		Completed university or college degree or higher	71 (44.9)	81 (45)	
	**ASGSRS^h,i^, n (%)**				
		Major cities and inner regional	100 (64.1)	157 (87.2)	
		Outer regional	56 (35.9)	26 (14.4)	

^a^ECEC: early childhood education and care.

^b^EPAO: Environment and Policy Assessment Observation.

^c^Missing data (control: n=1).

^d^Missing data (intervention: n=15; control: n=10).

^e^SEIFA: Socioeconomic Indexes for Areas.^f^Missing data (intervention: n=9; control n=7).

^g^Missing data (intervention: n=7; control: n=10).

^h^ASGSRS: Australian Statistical Geography Standard Remoteness Structure.

^i^Missing data (intervention: n=9; control: n=10).

### Packing and Consumption of Foods From Lunch Boxes

The results for the primary outcomes after multiple imputation and for complete case analysis are presented in Table 2. There was no significant difference between groups for the primary outcomes (kilojoules from discretionary foods and kilojoules from saturated fat, free sugars, and grams of sodium from all foods) packed or consumed. In addition, there was no difference in secondary outcomes, including packed or consumed serves of any core food groups or discretionary food serves between groups (Table 3). Data regarding the nutrients and food groups consumed are presented in Multimedia Appendix 5. The sensitivity analysis, which included only those parents known to have downloaded the app (181/355, 51%), also did not detect any statistically significant differences (Multimedia Appendix 6). The subgroup analysis by gender and SES demonstrated no significant differences between groups (Multimedia Appendix 7).

**Table 2:** Mean change in total energy, energy from discretionary foods, and associated nutrients by group (packed).

Energy and nutrients			Intervention (n=139)				Control (n=167)					Imputed difference postintervention^a^					Complete case analysis^a^		
			Baseline, mean (SD)		Postintervention, mean (SD)	Baseline, mean (SD)			Postintervention, mean (SD)		Mean difference (95% CI)			*P* value^b^		Mean difference (95% CI)			*P* value^b^
**Packed**																			
	Total energy (kJ)	2892.68 (850.71)		2820.13 (774.26)		2077.76 (672.89)		2762.47 (829.61)		39.53 (−264.57 to 343.64)			.80		19.45 (−293.05 to 331.94)			.90	
	Energy from discretionary foods (kJ)	774.58 (644.77)		799.26 (693.29)		800.78 (724.92)		712.03 (685.86)		77.84 (−163.49 to 319.18)			.53		62.78 (−190.87 to 316.42)			.60	
	Saturated fat (g)	9.64 (4.79)		8.62 (4.76)		8.97 (5.03)		7.84 (4.43)		0.72 (−0.52 to 1.96)			.26		0.56 (−1.00 to 2.12)			.45	
	Free sugars (g)	13.48 (9.65)		14.32 (11.94)		14.32 (11.94)		12.30 (11.59)		2.27 (−0.14 to 4.68)			.06		1.04 (−2.23 to 4.32)			.50	
	Sodium (mg)	1010.17 (426.86)		986.98 (365.59)		1026.12 (420.24)		934.74 (390.30)		49.93 (−68.86 to 168.72)			.41		60.78 (−95.85 to 217.42)			.42	

^a^All data adjusted for baseline and clustering and service Environment and Policy Assessment Observation score at baseline.

^b^Statistical significance inferred by *P* values <.01.

**Table 3:** Mean change in serves of discretionary foods and core food groups packed by group.

Food group			Intervention (n=139)					Control (n=167)					Imputed difference postintervention^a^					Complete case analysis^b^		
			Baseline, mean (SD)		Postintervention, mean (SD)		Baseline, mean (SD)			Postintervention, mean (SD)		Mean difference (95% CI)			*P* value^c^		Mean difference (95% CI)			*P* value^c^
**Packed (serves)**																				
	Discretionary foods^d^	1.29 (1.07)		1.33 (1.16)		1.33 (1.16)			1.19 (1.14)		0.13 (−0.27 to 0.53)			.53		0.10 (−0.32 to 0.53)			.60	
	Breads and cereals^e^	2.03 (1.02)		2.10 (1.01)		2.17 (0.96)			2.30 (1.06)		−0.27 (−0.58 to 0.05)			.09		−0.21 (−0.51 to 0.09)			.15	
	Fruit^f^	1.27 (0.85)		1.22 (0.82)		1.31 (0.89)			2.30 (1.06)		−0.10 (−0.47 to 0.27)			.61		−0.12 (−0.50 to 0.26)			.59	
	Vegetables^g^	0.25 (0.40)		0.20 (0.36)		0.21 (0.38)			0.21 (0.38)		0.00 (−0.10 to 0.10)			.97		−0.02 (−0.13 to 0.08)			.63	
	Dairy^h^	0.71 (0.52)		0.61 (0.49)		0.57 (0.50)			0.57 (0.50)		0.02 (−0.12 to 0.17)			.75		0.00 (−0.14 to 0.15)			.98	
	Meat and alternatives^b^	0.06 (0.21)		0.06 (0.20)		0.07 (0.23)			0.05 (0.16)		0.01 (−0.04 to 0.06)			.60		0.00 (−0.05 to 0.06)			.87	

^a^All data adjusted for baseline and clustering and service Environment and Policy Assessment Observation score at baseline.

^b^Meat and alternatives: examples of 1 serve=65 g of cooked lean meat, 80 g of cooked poultry, 100 g cooked fish, 2 large eggs, 1 cup of legumes or beans [6].

^c^Statistical significance inferred by *P* values <.01.

^d^Calculated using 600 kJ equivalents; that is, approximately 2 scoops of ice cream, 50 to 60 g of processed meats, 30 g of salty crackers, 2 to 3 sweet biscuits, and 1 (40 g) donut [6].

^e^Breads and cereals: examples of 1 serve=1 slice of bread; half medium roll; half cup of cooked rice, pasta, or noodles; and two-third cup wheat cereal flakes [6].

^f^Vegetables: examples of 1 serve=half cup cooked vegetables; half cup beans, peas, or lentils; 1 cup of leafy green or raw vegetables; half medium potato; and 1 medium tomato [6].

^g^Fruit: examples of 1 serve=1 medium apple, 2 small fruits, 1 cup of diced or canned fruit, and 30 g of dried fruit [6].

^h^Dairy and alternatives: examples of 1 serve=1 cup milk, 2 slices of hard cheese (40 g), and three-fourth cup yogurt [6].

### Process Evaluation

#### Intervention Fidelity

All (11/11, 100%) of the push notifications messages were delivered via the app to intervention ECEC services as planned. A message video link failed at week 9 and was resent as part of an unplanned additional message in week 11. All service managers reported sending the preintervention and midway planned support messages to parents (7/7, 100%; missing data 1/7, 14%).

#### App Downloads and SWAP IT Program Use

All ECEC services had access to the app, and it was available for all parents to download. The percentage of intervention families with data at baseline (as included in the multiple imputation analysis) and known to have the intervention app was 61.8% (102/165; missing data on app ownership 15/165, 9.1%).

Data on the number of unique within-app message views are presented in Multimedia Appendix 4 [50]. As app analytics only provided deidentified data, data specific to parents in the trial could not be separated from data on all users of the app at the intervention ECEC services. The number of unique within-app message views decreased over time, with a mean of 139 (SD 42.7) views per message and a mean viewing rate of 26% (SD 14.9%).

#### SWAP IT Program Acceptability

Data on the acceptability of the *SWAP IT* program by service managers and parents are reported in Multimedia Appendix 4 [50]. Parents who completed the survey reported that they liked the *SWAP IT* program (34/41, 83%) and found the program useful (33/41, 80%) and easy to use (36/41, 87%). Only 57% (4/7) of ECEC services rated the overall program as useful; however, most agreed that the resources within the program were helpful for families (6/7, 86%). Both ECEC services (8/8, 100%) and families that completed the survey (41/41, 100%) agreed or had no feelings either way regarding the timing and frequency of the push notification measures.

#### Feasibility of Ongoing Use of the App

Most ECEC services and parents who completed the survey agreed that it was appropriate to deliver lunch box information via the app (6/8, 75% and 40/50, 80%, respectively). During the intervention, most intervention ECEC services reported using the app for functions other than the delivery of the program (6/7, 86%; eg, for distribution of parent newsletters). Self-reported use of the app ranged from 0 to 2 hours per week. Only 57% (4/7) of ECEC services indicated that they planned to continue to use the app, with 29% (2/7) of ECEC services indicating they were unsure and 14% (1/7) of services reporting that they did not plan to continue with the app after the end of the SWAP IT program.

#### Cointervention and Adverse Events

The service reported changes in the frequency of parent complaints or concerns regarding healthy lunch box policy did not differ between the groups. No contamination was reported; that is, the app was not used to send any other health or nutrition information. No ECEC services in the intervention or control groups reported exposure to additional nutrition interventions throughout the duration of the trial.

#### Outcomes From the Study Protocol Not Reported

Owing to the null findings, neither a cost-effectiveness analysis, as specified in the study protocol, nor the planned analysis of data collected on the usual daily dietary intake of children (to be able to detect any compensatory dietary behaviors) was conducted.

#### Association Between ECEC Service Hours of Routine Use of the App and Parent Viewing Rate

The exploratory analysis found an association between the number of routine hours of use of the app by ECEC services and the parent viewing rate of within-app messages; however, this association was not significant (*P*=.21; data not provided).

## Discussion

### Principal Findings

To our knowledge, the *SWAP IT Childcare* trial is the first trial to evaluate the efficacy of an mHealth intervention to improve lunch box contents in ECEC settings internationally. However, contrary to our hypothesis, the intervention had no impact on the primary outcome of the number of kilojoule from discretionary foods packed in children’s lunch boxes. In addition, process evaluation found low parent app downloads and message viewing rates.

When looking to compare the findings of the trial with other published studies, we were unable to identify any other healthy eating–focused mHealth interventions or DHIs targeting parents and children through ECEC settings. Therefore, we have primarily compared the findings with those of the original *SWAP IT* trial publications conducted in the primary school setting [39].

Although the results of our parent survey data indicated that delivery of a lunch box program via the app was considered appropriate, the mean viewing rate of the within-app message was only 26% (SD 14.9). Furthermore, only 61.8% (102/165) of parents in the intervention group were known to have downloaded the app, leaving 38.2% (63/165) of parents unexposed to program content. Such process data are in contrast to findings reported in a *SWAP IT* pilot study undertaken in a primary (elementary) school setting [39]. The intervention in the *SWAP IT* pilot study was similar to this study, including push notifications and within-app message content; however, it also included the distribution of resources to parents, policy, and classroom resources [39]. The pilot study reported higher parent app downloads (89%) and message views (between 35% and 120% over the 10-week intervention) and was able to demonstrate a reduction in the packing of kilojoules from discretionary foods (−221 kJ; *P*=.08, nonsignificant) [39].

Contextual differences between the ECEC and school settings in the study region may potentially explain the differences in the findings between these trials. In the school trial, all schools were existing users of the app used to deliver the *SWAP IT* program compared with just 2 ECEC services in the intervention arm of this trial. In addition, most parents were existing users of the app (355/400, 88.8%) [39]. Greater integration and preprogram use of the app may have meant that parents in the school trial were more likely to be routinely accessing the app to receive information and, in doing so, were more likely to access the *SWAP IT* program content. This premise is supported by our exploratory analysis, which found a positive nonsignificant association between how much time the ECEC services used the app for general communication with parents and the *SWAP IT* parent message viewing rate. In addition, it is possible that the inclusion of supportive ECEC service–based strategies targeting nutrition curriculum and policy, similar to that undertaken to the trial in schools, may have been useful in increasing the impact of the intervention.

Our ability to detect an effect on discretionary food packing may have also been limited by the lower than anticipated baseline packing of discretionary foods in the participating ECEC services [48]. The trial baseline data found ECEC child lunch boxes contained only a mean of 1.33 (SD 1.16) serves of discretionary foods [48]. This was considerably lower than the mean of 2.5 serves per lunch box reported in schools in the same region [55]. Although the serves of discretionary foods packed in the ECEC services exceeded recommended serves per day for our age group (ie, 0-0.5 serves) [6], the lower number of serves packed at baseline possibly limited scope for further improvement without a more intensive intervention approach. In contrast, a large scope for improving vegetable packing and consumption in children attending ECEC services was evident from the trial baseline data (only 0.3 mean vegetable serves packed and 0.1 serves consumed) [48]. As such, future iterations of the intervention would benefit from the inclusion of specific strategies to improve vegetable packing and intake, along with continued efforts to minimize discretionary foods.

The intervention’s lack of impact on parent packing of discretionary foods may have also been because of the decline in the rate of program message opening over the course of the trial. The pattern of the continued drop-off in use observed in our study is similar to those reported in many mHealth and DHI interventions [39,56-59]. The drop-off in engagement occurred in the trial despite the inclusion of several features explicitly designed to enhance engagement (user consultation, use of behavior change techniques, reminders, and information being provided from a credible source) [60,61] and the addition of a specific intervention strategy to prompt ongoing use (service manager delivered support messages). Additional factors found to be associated with sustained DHI use in the literature include perceived ongoing relevance, novelty value, tailoring, and self-monitoring [60,62]. Further qualitative exploration of parents’ reasons for low engagement and consideration of additional evidence-based features that could be added to the program to enhance engagement would be beneficial.

### Strengths and Limitations

The strengths of this study include explicit mapping of behavior change theory to design the intervention, use of objective gold standard measures to assess lunch box contents, high follow-up rate, comprehensive process measures, and randomized trial design. A key limitation of the trial is that the app analytics could only capture use data at the service level, meaning that the true proportion of parents who accessed the messages is unknown (as message views may have been from parents from the service but not participating in the evaluation component of the trial). This limited our ability to conclusively determine whether the null findings were generally more likely to be a result of poor access, the program content itself, or a combination. This limitation extends to our additional analysis of only those parents who were known to have downloaded the app. Finally, given the low survey response rate (41/190, 21.6%) and differences in characteristics in survey completers compared with the overall parent sample, our acceptability survey data may have been influenced by response bias. The generalizability of the findings to ECEC services outside of the study region may be limited.

### Conclusions

The intervention failed to decrease energy from discretionary foods in children’s lunch boxes. However, the use of apps was rated as an appropriate modality for delivering information related to the packing of healthier foods in lunch boxes. Process data suggest that the lack of impact may have been because of factors associated with the implementation of the intervention, such as the low parent uptake and use of the app, as well as the lower than expected levels of packing of discretionary foods at baseline. Given these limitations, the current feasibility of using mHealth interventions to target parent packing of healthier foods in lunch boxes is still uncertain. Future trials should invest time in collecting formative data on how ECEC services and parents currently use and engage with different digital platforms to best identify the ideal technology to deliver parent-focused nutrition interventions in this setting. In addition, given drop-offs in use over time, exploration of parent reasons behind poor ongoing use will also assist in enhancing program design and content to improve engagement.

## Appendix

Multimedia Appendix 1 CONSORT-EHEALTH checklist V 1.6.1.

Multimedia Appendix 2 Use of behaviour change techniques.

Multimedia Appendix 3 Adaptation process.

Multimedia Appendix 4 Process evaluation.

Multimedia Appendix 5 Secondary outcome data.

Multimedia Appendix 6 Sensitivity analysis.

Multimedia Appendix 7 Subgroup analysis.

## Abbreviations

BCW: behavior change wheel
CONSORT-EHEALTH: Consolidated Standards of Reporting Trials of Electronic and Mobile Health Applications and online Telehealth
DHI: digital health intervention
ECEC: early childhood education and care
EPAO: Environment and Policy Assessment Observation
HNE: Hunter New England
mHealth: mobile health
NSW: New South Wales
SES: socioeconomic status
