# Supplementary material for: An mHealth Intervention to Reduce the Packing of Discretionary Foods in Children’s Lunch Boxes in Early Childhood Education and Care Services: Cluster Randomized Controlled Trial
Source: J Med Internet Res. 2022 Mar 17;24(3):e27760. doi: 10.2196/27760 (PMC8972115; doi:10.2196/27760)
Supplement: Multimedia Appendix 2 [file jmir_v24i3e27760_app2.docx]

Multimedia Appendix 2: Use of Behavioural Change techniques (BCTs) in the intervention

| BCTs | Integration | Aim/ purpose |
| --- | --- | --- |
| Prompts/ cues | Push notifications alerting parents to in-app messages regarding healthy lunchboxes | To serve as a weekly reminder to parents to pack healthy foods in the lunchbox  To prompt parents to access information to assist them to overcome potential barriers to packing healthy lunchboxes |
| Verbal persuasion about capability | Wording and language used in fact sheets and webpages designed to convey support and provide practical tips for overcoming barriers to packing healthy lunchboxes for example “Trying to get your child to eat new foods can be challenging, however it’s important to remember that most fussy eaters grow into older children who enjoy a wide variety of foods.” | To reduce parent self-doubt and increase belief and confidence in ability to overcome common barriers to packing healthy foods |
| Behaviour substitution | The focus of the intervention was on “swapping” less healthy foods for healthier choices. Parents were provided specific examples of common discretionary foods packed in lunchboxes, as well as an idea for a healthier alternative. An example of a healthy swap was presented visually as a graphic in each within-app message and as part of fact sheets on ideas for savoury and sweet snack “swaps”. The SWAP IT Options section of the website included specific examples of both foods recommended to “swap out” and “swap in”. | To provide specific examples for healthier foods to replace discretionary foods in the lunchbox. This was designed to overcome the barriers of lack of knowledge and lack of ideas for what to pack. |
| Instruction on how to perform the behaviour | Parents were provided with advice and examples of foods recommended to include in healthy lunchboxes as part of the content of the within-app messages and supporting resources. | To increase parent knowledge of recommended foods and non-recommended foods and new ideas for the lunchbox. |
| Conserving mental resources | The “SWAP IT Options” section of the website provides a comprehensive list of foods suitable for the lunchbox, including product brands and flavours. Parents could simply look up suitable lunchbox food items from list to purchase for lunchboxes. | To reduce mental burden by overcoming the need to decipher nutrition labels and help distinguish between healthy and less healthy food items. |
| Information about health consequences | A fact sheet and within app message targeted how excessive intake of discretionary foods may displace healthy, nutrient dense foods in children’s diets and have an impact on health. | To increase parent awareness of the health benefits of reducing the amount of discretionary foods in the lunchbox. This aimed to assist with targeting the belief that it is not important to reduce packing of discretionary food. |
| Demonstration of the behaviour | Links to videos were included in some within-app messages and were used to demonstrate behaviours such packing a healthy lunchbox when short on time, techniques to support children to try new foods, and a shopping demonstration on how you can save money when buying healthy foods. | To increase feelings of capability and self-efficacy in parents experiencing barriers to packing healthy lunchboxes including lack of time, cost and dealing with fussy eaters. |
