# Supplementary material for: An mHealth Intervention to Reduce the Packing of Discretionary Foods in Children’s Lunch Boxes in Early Childhood Education and Care Services: Cluster Randomized Controlled Trial
Source: J Med Internet Res. 2022 Mar 17;24(3):e27760. doi: 10.2196/27760 (PMC8972115; doi:10.2196/27760)
Supplement: Multimedia Appendix 3 [file jmir_v24i3e27760_app3.docx]

Multimedia Appendix 3: Adaptation process based on the FRAME framework

| Original SWAP IT Program Component (primary schools setting) | Goal/ Purpose of adaptation | Process (by whom) | Outcome | Adaptations |
| --- | --- | --- | --- | --- |
| Provision of weekly push notifications and within-app messages addressing barriers to packing healthy lunchbox foods.  Within message links to support material (website) and videos | Improved fit with participants | Identification of ECEC specific parent barriers to packing healthy lunchboxes (ECEC research team survey of a sample of ECEC parents, consultation with ECEC services, cultural consultation, literature)  Review of existing support information on the website and video content (research team with expertise in delivery of nutrition programs to ECEC settings) | New barriers were identified and one previous barrier was deemed not applicable to the ECEC setting (food safety concerns).  The need for a greater emphasis on fussy eating behaviours was identified as a significant barrier to packing healthy food.  Some website content was found not to be compatible or relevant for the ECEC setting. Some existing videos contained school specific references and contained examples of lunchboxes not typical packed for children in the ECEC age group. | Weekly push messages and within-app message content adapted to address ECEC specific barriers  Additional section of the website developed specific to ECEC  Development and addition of two new videos to increase relevance to ECEC setting. |
| Provision of “SWAP IT Options” (online lists of foods recommended and not recommended for the lunchbox) | Improved fit with participants | Review of existing SWAP IT lists of food recommended for school children. (Dietitians and health promotion officers within ECEC research team). | Some foods identified that may not be developmentally appropriate for ECEC-aged children. There was also potential to expand the list to include more foods commonly consumed by ECEC children. | The SWAP IT lists developed for the school intervention was adapted to include additional foods typically consumed by ECEC-aged children and provide warnings for potential choking risk foods. |
| Endorsement of program by School Principal | Improved fit with participants  Improved retention | Consultation with the schools-based research team regarding endorsement and factors that may affect ongoing engagement (ECEC research team) | Securing executive support to be maintained as a strategy.  As parents read messages less over time, ECEC service manager support may be leveraged to enhance parents ongoing participation thought the trial. | Researchers met with intervention service managers to secure executive support prior to the commencement of the program.  Service managers were asked to promote ongoing parent participation in the program (i.e. accessing the in-app messages) as an additional strategy. |
| Development and provision of School Nutrition Guidelines | Improved fit with participants/ setting | Review of need for the strategy (ECEC research team) | ECEC services known to already have nutrition and lunchbox related policy | Not included |
| Lunchbox flipchart lessons | Improved fit with participants  Reduce costs | Review of need for the strategy via consultation with schools research team (ECEC research team)  Research team prioritisation of strategies due to limited funds available for trial (ECEC-based research team) | Strategy was not considered a core component | Not included |
| Additional Resources (booklet, ice brick, water bottle) | Improved fit with participants  Reduce costs | Review of need for the strategy via consultation with schools research team (ECEC research team) | The ECEC research team already routinely distributes similar booklets to ECEC services in the region as part of ongoing health promotion programs  The ice brick was provided to address the school-specific barrier of parent safety concerns however this was not a barrier identified by ECEC services (ECEC services provide refrigeration for lunchboxes)  ECEC already have guidelines around provisions of sweetened drinks and therefore water bottles were not thought to be a priority for the ECEC setting. | Not included |
