# Supplementary material for: An mHealth Intervention to Reduce the Packing of Discretionary Foods in Children’s Lunch Boxes in Early Childhood Education and Care Services: Cluster Randomized Controlled Trial
Source: J Med Internet Res. 2022 Mar 17;24(3):e27760. doi: 10.2196/27760 (PMC8972115; doi:10.2196/27760)
Supplement: Multimedia Appendix 4 [file jmir_v24i3e27760_app4.docx]

**Multimedia Appendix 4: Process Evaluation**

1. Process Outcome measures

Table 1.1 Measures Summary

| Measure | Details |
| --- | --- |
| Intervention Fidelity | App analytics were used to determine the proportion of push notification messages delivered as planned. The proportion of planned service manager communications sent (via the app or email in order to support parents during the intervention) was measured via a self-completed written record of implementation distributed to service managers pre-intervention and collected at post-intervention follow up data collection. |
| App downloads and SWAP IT program usage | The number of consenting parents who had downloaded the app was estimated from the initial consent forms and combined with ECEC service generated reports. Estimates of parent use of the program (de-identified data available on number of unique within-app message views) was collected via app analytics. Estimates of service viewing rates were calculated using total unique within-app message service views divided by number of families enrolled at each service, multiplied by 100. |
| SWAP IT program acceptability | Acceptability (n= 17) questions measuring if the intervention was agreeable and useful were included as part of a wider online survey emailed to parents in the intervention group, post- intervention. Additional details of the online survey reported in accordance with the CHERRIES checklist are as a separate Multimedia Appendix [Multimedia Appendix 5: CHERRIES Checklist]. Service manager acceptability of the intervention was assessed via a pen and paper survey. Both parent and service questions were modified versions of items taken from the Acceptability of Intervention Measure (AIM).[50] |
| Feasibility of ongoing use of the App (i.e. actual fit or practicability) and appropriateness (i.e. perceived fit or relevance). | Two questions included in the post-intervention service manager survey. Questions were adapted from the Feasibility of Intervention Measure. [1] ECEC services were asked if the app was utilised by the service for any other purpose during the trial (e.g. routine communications with parents regarding service events), how much time per week they used the app and if the service planned to continue using the app post-trial.  To assess appropriateness of the intervention the parent online survey included a question asking if they thought it was appropriate to receive lunchbox information via the app. Both the service manager survey and online parent survey questions utilised a five-point Likert scale (strongly agree, agree, no strong feelings either way, disagree, strongly disagree). |
| Co-intervention and adverse events | The post service manager pen and paper survey asked if the service had participated in any other nutrition intervention and about the type of additional content they communicated through the app during the intervention period. To capture any increase in adverse events as a result of the intervention, ECEC services were asked the estimate the general frequency of parent complaints regarding lunchbox guidelines in both the pre and post service manager survey. |

2. Process Evaluation Data

Table 2.1 Unique within in-app message views per week

| Weekly within-app message (accessible via weekly push notification alert or within app content) | Total unique weekly within-app message views^a^ |
| --- | --- |
| Week 1, Message 1: “Welcome to the SWAP IT Lunchbox Program”  Week 1, Message 2: “The ultimate list of healthy lunchbox foods” | 186  202 |
| Week 2: “5 *sweet* ideas for the lunchbox” | 169 |
| Week 3: “How to save $$ when packing the lunchbox” | 163 |
| Week 4: “Worried about your fussy eater? Read this!” | 155 |
| Week 5: “Healthy savoury snacks that are a hit” | 138 |
| Week 6: “Why are some lunchbox snacks better than others?” | 139 |
| Week 7: “Drinking water” | 121 |
| Week 8: “Our top 5 lunch box time saving hacks” | 107 |
| Week 9: “Fussy eaters: Supporting children to try new foods” | 60 |
| Week 10: “Thanks for being part of SWAP IT” | 87 |

^a^Unique view data as available from the app analytics. Numbers can be higher than

intervention group totals as messages were accessible by all families at the intervention

services.

Table 2.2 Mean viewing rate per service

| ECEC Intervention Services (N= 8) | Number of families enrolled per service | Mean message viewing rate^a^ |
| --- | --- | --- |
| A | 65 | 23 |
| B | 59 | 37 |
| C | 106 | 26 |
| D | 27 | 30 |
| E | 61 | 2 |
| F | 80 | 39 |
| G | 54 | 6 |
| H | 73 | 42 |

^a^ Mean number of views per week as a percentage of estimated potential views (i.e. number of families enrolled at the service).

Table 2.3 Parent and Service Acceptability

| Parent^a^ (N= 41) | Strongly Agree/ Agree  % (N) | No strong feelings either way  % (N) | Disagree/ strongly disagree  % (N) |
| --- | --- | --- | --- |
| Overall, I found the SWAP IT program useful | 80 (33) | 17 (7) | 2 (1) |
| Overall, I liked the SWAP IT program | 83 (34) | 17 (7) | 0 (0) |
| I found the information easy to use | 87 (36) | 12 (5) | 0 (0) |
| I liked the layout/ look of the SWAP It program | 83 (34) | 17 (7) | 0 (0) |
| I found the messages helpful | 76 (31) | 24 (10) | 0 (0) |
| Found the number of messages appropriate | 49 (20) | 51 (21) | 0 (0) |
| I was happy with the time of day the messages were delivered (1.00pm on Thursdays) | 46 (19) | 54 (22) | 0 (0) |
| It is appropriate to receive health information via the existing app^b^ | 80 (40) | 20% (10) | 0 (0) |
| Service (N= 8) | Strongly Agree/ Agree  % (N) | No strong feelings either way  % (N) | Disagree/ strongly disagree  % (N) |
| Found the SWAP IT program useful^c^ | 57 (4) | 43 (3) | 0 (0) |
| Believed the SWAP IT program influenced the kind of food families packed in the lunchbox | 57 (4) | 29 (2) | 14 (1) |
| Believed the resources and information (i.e. the messages, fact sheets, weblinks, videos) provided as part of SWAP IT were helpful for families^c^ | 86 (6) | 14 (1) | 0 (0) |
| Believed the SWAP IT Choices lists (online list of foods suitable for the lunchbox) were helpful for families | 88 (7) | 12 (1) | 0 (0) |
| Believe the existing app was a suitable way to deliver healthy lunchbox information to families | 75 (6) | 25 (2) | 0 (0) |
| Believe the timing and frequency of push notification messages delivered as part of the SWAP IT program was suitable (10 push messages one per week over 10 weeks, delivered on Thursdays at 1.00pm). | 75 (6) | 25 (2) | 0 (0) |

^a^Intervention parents who responded to the online survey and answered “yes” to having accessed any of the SWAP IT content

^b^ Survey responders reporting to have the app and used the app during SWAP IT (N= 50)

^c^ Missing data N= 1

Reference

[50] Weiner BJ, Lewis CC, Stanick C, Powell BJ, Dorsey CN, Clary AS, et al. Psychometric assessment of three newly developed implementation outcome measures. Implementation science: IS. 2017 08;12(1):108. PMID: 28851459. doi: 10.1186/s13012-017-0635-3.
