# Supplementary material for: An mHealth Intervention to Reduce the Packing of Discretionary Foods in Children’s Lunch Boxes in Early Childhood Education and Care Services: Cluster Randomized Controlled Trial
Source: J Med Internet Res. 2022 Mar 17;24(3):e27760. doi: 10.2196/27760 (PMC8972115; doi:10.2196/27760)
Supplement: Multimedia Appendix 5 [file jmir_v24i3e27760_app5.docx]

Multimedia Appendix 5: Secondary Outcome data

Table 1: Mean change in total energy, energy from discretionary foods and associated nutrients by group (consumed)

|  | Intervention | | Control | | Imputed difference  post-intervention^b^ | | Complete case analysis^b^ | |
| --- | --- | --- | --- | --- | --- | --- | --- | --- |
|  | Baseline  Mean (SD) | Post- intervention  Mean (SD) | Baseline  Mean (SD) | Post-intervention  Mean (SD) | Mean difference (CI) | P value^a^ | Mean difference  (CI) | P value^a^ |
| Consumed | N= 138 | N= 137 | N=165 | N= 164 |  |  |  |  |
| Mean total energy (kJ) | 2077.37 (619.58) | 2055.50 (715.45) | 1908.03 (669.15) | 1896.07 (682.29) | 137.85 (-81.21; 356.91) | .22 | 120.96  (-71.10 ; 313.02) | .20 |
| Mean energy from discretionary foods (kJ) | 542.72 (490.80) | 569.23 (586.49) | 476.44  (484.39) | 482.50 (531.75) | 103.35 (-75.97; 282.67) | .26 | 87.76  (-95.91 ; 271.43) | .32 |
| Mean saturated fat (g) | 7.10 (3.97) | 6.35 (3.91) | 6.01 (3.72) | 5.66 (3.6) | 0.72 (-0.52; 1.96) | .26 | 0.56  (-0.64 ; 1.77) | .33 |
| Mean free sugars (g) | 10.04 (7.24) | 10.80 (9.87) | 9.19 (8.15) | 8.27 (8.14) | 2.27 (0.14; 4.68) | .06 | 2.22  (0.28 ; 4.72) | .08 |
| Mean sodium (mg) | 769.80 (336.47) | 749.15 (307.40) | 697.25 (334.04) | 682.48 (327.59) | 49.93 (-68.86-168.72) | .41 | 61.89  (-47.67 ; 171.59) | .24 |

^a^Statistical significance inferred by P values < 0.01

^b^All data adjusted for baseline and clustering and service EPAO score at baseline.

Table 2: Mean change in serves of discretionary foods and core food groups consumed by group

|  | Intervention | | Control | | Imputed difference  post-intervention^b^ | | Complete case analysis^b^ | |
| --- | --- | --- | --- | --- | --- | --- | --- | --- |
|  | Baseline  Mean (SD) | Post-intervention  Mean (SD) | Baseline  Mean (SD) | Post- intervention  Mean (SD) | Mean difference (CI) | P value^a^ | Mean difference  (CI) | P value^a^ |
| Consumed (serves) | N=138 | N=137 | N=165 | N=164 |  |  |  |  |
| Discretionary foods^c^ | 0.90 (0.82) | 0.95 (0.98) | 0.79 (0.81) | 0.80 (0.89) | 0.17 (-0.13; 0.47) | .26 | 0.15  (-0.16 ; 0.45) | .32 |
| Breads and cereals^d^ | 1.54 (0.87) | 1.62 (0.91) | 1.62 (0.91) | 1.61 (1.03) | -0.10 (-0.39; 0.20) | .53 | -0.06  (-0.34 ; 0.23) | .68 |
| Fruit^e^ | 0.78 (0.64) | 0.79 (0.66) | 0.70 (0.65) | 0.79 (0.69) | 0.03 (-0.20; 0.27) | .78 | 0.01  (-0.23 ; 0.22) | .93 |
| Vegetables^f^ | 0.15 (0.30) | 0.13 (0.26) | 0.14 (0.37) | 0.11 (0.24) | 0.03 (-0.20; 0.27) | .78 | 0.02 (-0.06; 0.09) | .65 |
| Dairy^g^ | 0.54 (0.45) | 0.48 (0.44) | 0.47 (0.42) | 0.44 (0.43) | 0.05 (-0.11; 0.21) | .53 | 0.03  (-0.14 ; 0.20) | .70 |
| Meat and alternatives^h^ | 0.05 (0.16) | 0.03 (0.13) | 0.05 (0.21) | 0.03 (0.10) | 0.00 (-0.3; 0.04) | .87 | 0.00 (-0.04; 0.03) | .92 |

^a^Statistical significance inferred by P values < 0.01

^b^All data adjusted for baseline and clustering and service EPAO score at baseline.

^c^ Calculated using 600kJ equivalents. I.e. approximately: 2 scoops of ice cream, 50-60g of processed meats, 30g of salty crackers, 2-3 sweet biscuits, 1 (40g) doughnut (6)

^d^ Breads and Cereals: Examples of 1 serve = 1 slice of bread, ½ medium roll, ½ cup of cooked rice, pasta or noodles, 2/3 dup wheat cereal flakes (6)

^e^ Vegetables: Examples of 1 serve = ½ cup cooked vegetables, ½ cup beans, peas or lentils, 1 cup of leafy green or raw vegetables, ½ medium potato, 1 medium tomato (6)

^f^ Fruit: Examples of 1 serve = 1 medium apple, 2 small fruits, 1 cup or diced or canned fruit, 30g of dried fruit (6)

^g^ Dairy and alternatives: Examples of 1 serve = 1 cup milk, 2 slices of hard cheese (40g), ¾ cup yoghurt (6)

^h^ Meat and alternatives: Examples of 1 serve = 65g of cooked lean meat, 80g of cooked poultry, 100g cooked fish, 2 large eggs, 1 cup of legumes/ beans (6)
