# Supplementary material for: An mHealth Intervention to Reduce the Packing of Discretionary Foods in Children’s Lunch Boxes in Early Childhood Education and Care Services: Cluster Randomized Controlled Trial
Source: J Med Internet Res. 2022 Mar 17;24(3):e27760. doi: 10.2196/27760 (PMC8972115; doi:10.2196/27760)
Supplement: Multimedia Appendix 6 [file jmir_v24i3e27760_app6.docx]

Multimedia Appendix 6: Sensitivity Analysis

Mean change in total energy, energy from discretionary foods and associated nutrients by group: only parents with the app

|  | Intervention | | Control | | Complete case analysis^a^ | *P* value*^b^* |
| --- | --- | --- | --- | --- | --- | --- |
|  | Baseline  Mean (SD) | Follow up  Mean (SD) | Baseline  Mean (SD) | Follow up  Mean (SD) | Mean difference  (CI) |  |
| *Packed* | N= 102 | N=102 | N=79 | N=79 |  |  |
| Mean total energy (kJ) | 3016.04 (799.68) | 2873.64 (768.65) | 2874.06 (831.93) | 2822.02 (825.91) | -56.70 (-413.32 ; 299.93) | .74 |
| Mean energy from discretionary foods (kJ) | 852.07  (654.71) | 866.78 (724.80) | 760.35 (680.93) | 767.35 (713.88) | -1.98 (-343.87 ; 339.90) | .86 |
| Mean saturated fat (g) | 9.97 (4.46) | 9.17 (5.00) | 8.24 (4.58) | 8.22 (4.53) | 0.67(-1.67 ; 3.01) | .42 |
| Mean free sugars (g) | 14.60 (9.91) | 15.01 (11.82) | 13.91 (12.06) | 13.02 (SD=11.12) | 0.90 (-3.09; 4.90) | .68 |
| Mean sodium (mg) | 1018.76 (392.66) | 997.36 (349.12) | 1006.58 (359.38) | 965.80 (401.15) | 30.32 (-147.64; 208.04) | .72 |
| *Consumed* | N= 102 | N= 100 | N=78 | N= 79 |  |  |
| Mean total energy (kJ) | 2141.56 (598.56) | 2119.21 (695.52) | 1874.53 (640.6) | 1863.70 (711.11) | 170.98 (-61.11; 403.07) | .14 |
| Mean energy from discretionary foods (kJ) | 602.40 (510.37) | 628.93 (625.03) | 477.99 (488.27) | 481.73 (543.14) | 123.00 (-89.35; 335.35) | .23 |
| Mean saturated fat (g) | 7.24 (3.83) | 6.76 (3.92) | 5.38 (3.05) | 5.76 (3.85) | 0.56 (-0.76 ; 1.88) | .38 |
| Mean free sugars (g) | 10.69 (7.48) | 11.46 (10.57) | 9.71 (9.13) | 8.40 (9.09) | 2.59 (-0.51; 5.68) | .17 |
| Mean sodium (mg) | 770.29 (324.06) | 769.91 (291.77) | 688.05 (332.80) | 675.13 (332.80) | 84.84 (-42.10 ; 211.79) | .17 |

^a^All data adjusted for baseline and clustering and service EPAO score at baseline

^b^Statistical significance inferred by P values < 0.01
