# Supplementary material for: An mHealth Intervention to Reduce the Packing of Discretionary Foods in Children’s Lunch Boxes in Early Childhood Education and Care Services: Cluster Randomized Controlled Trial
Source: J Med Internet Res. 2022 Mar 17;24(3):e27760. doi: 10.2196/27760 (PMC8972115; doi:10.2196/27760)
Supplement: Multimedia Appendix 7 [file jmir_v24i3e27760_app7.docx]

Multimedia Appendix 7: Sub-group analysis

Sub-group analysis of primary outcomes (packed data) by sex and gender

| Outcome and sub-group | Intervention | | Control | | Complete case analysis^a^ | | Relative difference (CI) | *P* value^b^ |
| --- | --- | --- | --- | --- | --- | --- | --- | --- |
|  | Baseline  Mean (SD) | Follow up  Mean (SD) | Baseline  Mean (SD) | Follow up  Mean (SD) | Mean difference  (CI) | *P* value^b^ |  |  |
| *Mean total energy (kJ)* | | | | | | |  |  |
| Gender^c^ | | | | | | | 29.91 [-299.12; 358.94] | .85 |
| Male | 3080.97 (944.58) | 2950.38 (771.82) | 3023.12 (874.95) | 2884.01 SD=858.53 | 7.63 [-353.36 ; 368.61] | .96 |  |  |
| Female | 2719.71 (717.20) | 2695.38 (761.18) | 2743.57 (838.20) | 2647.99 (789.34) | 37.54  [-310.26 ; 385.34] | .82 |  |  |
| SEIFA^d^ | | | | | | | -71.40 [-583.68; 438.87] | .74 |
| Least disadvantaged | 2606.73 (816.88) | 2663.15 (744.13) | 2769.57 (827.28) | 2645.59 (766.39) | 45.78 [-408.92 ; 500.49] | .81 |  |  |
| Most disadvantaged | 3091.43 (844.76) | 2933.26 (762.15) | 2947.66 (877.09) | 2878.77 (882.77) | -26.62 [-450.78 ; 367.54] | .88 |  |  |
| *Mean energy from discretionary foods (kJ)* | | | | | | |  |  |
| Gender^c^ | | | | | | | -18.80  [-313.81 ; 276.06] | .89 |
| Male | 909.40 (669.06) | 876.20 (760.28) | 862.75 (715.75) | 764.80 (721.46) | 73.76  [-223.82; 371.34] | .61 |  |  |
| Female | 650.73 (599.09) | 725.56 (618.92) | 742.60 (715.75) | 662.33 (650.87) | 54.89  [-230.74; 340.51] | .69 |  |  |
| SEIFA^d^ | | | | | | | 16.78 [-432.37; 465.93] | .93 |
| Least disadvantaged | 591.17 (516.07) | 674.87 (565.58) | 778.97 (664.28) | 672.52 (618.58) | 41.70 [-350.60; 433.99] | 1.00 |  |  |
| Most disadvantaged | 895.08 (694.28) | 884.25 (759.88) | 778.97 (664.28) | 738.54 (751.44) | 58.47 [-306.11; 423.06] | .80 |  |  |
| *Mean saturated fat (g)* | | | | | | |  |  |
| Gender^c^ | | | | | | | .53 [-1.57 ; 2.62] | .60 |
| Male | 10.15 (4.88) | 8.77 (4.90) | 9.29 (5.62) | 8.21 (4.39) | 0.29 [-1.63 ; 2.20] | .75 |  |  |
| Female | 9.18 (4.69) | 8.48 (4.66) | 8.67 (4.42) | 7.50 (4.46) | 0.81 [-1.01 ; 2.64] | .36 |  |  |
| SEIFA^d^ | | | | | | | -1.96 [-4.83; 0.91] | .15 |
| Least disadvantaged | 8.73 (4.62) | 8.88 (5.34) | 8.66 (4.98) | 7.34 (4.29) | 1.62 [-0.75 ; 3.99] | .15 |  |  |
| Most disadvantaged | 10.27 (4.927) | 8.52 (4.50) | 9.20 (5.18) | 8.25 (4.63) | -0.34 [-2.54; 1.86] | .72 |  |  |
| *Mean free sugars (g)* | | | | | | |  |  |
| Gender^c^ | | | | | | | .81 [-4.14 ; 5.75] | .73 |
| Male | 15.16 (10.40) | 16.13 (13.51) | 14.45 (11.84) | 13.92 (13.66) | 0.62  [-3.61; 4.86] | .76 |  |  |
| Female | 11.93 (8.68) | 12.60 (10.02) | 12.58 (10.80) | 10.77 (9.04) | 1.43 [-2.59 ; 5.45] | .46 |  |  |
| SEIFA^d^ | | | | | | | 1.07 [-5.28 ; 7.41] | .65 |
| Least disadvantaged | 10.13 (7.41) | 11.17 (8.94) | 12.93 (10.91) | 11.62 (11.00) | -0.12  [-5.40; 5.16] | .96 |  |  |
| Most disadvantaged | 15.47 (10.44) | 16.30 (13.30) | 13.90 (11.54) | 13.03 (12.17) | 1.15 [-3.72; 6.03] | .58 |  |  |
| *Mean sodium (mg)* | | | | | | |  |  |
| Gender^c^ | | | | | | | -72.34 [-247.16 ; 102.48] | .39 |
| Male | 1103.52 (506.58) | 1044.29 (391.74) | 1067.18 (427.73) | 941.85 (362.24) | 100.67 [-80.61 ; 281.95] | .25 |  |  |
| Female | 924.41 (317.07) | 932.09 (332.21) | 987.58 (411.55) | 928.04 (417.02) | 28.33 [-145.81; 202.47] | .73 |  |  |
| SEIFA^d^ | | | | | | | -115.31 [-363.61; 133.00] | .30 |
| Least disadvantaged | 895.83 (361.89) | 947.33 (378.89) | 994.54 (440.12) | 860.95 (345.76) | 104.81.09 [-104.11; 313.73] | .27 |  |  |
| Most disadvantaged | 1082.42 (457.17) | 1003.55 (354.04) | 1043.19 (412.32) | 1000.24 (419.05) | -10.49 [-204.05; 183.07] | .90 |  |  |

^a^All data adjusted for baseline and clustering and service EPAO score at baseline

^b^Statistical significance inferred by P values < 0.01

^c^Intervention: Male, N= 68, Female, N= 71. Control: Male, N= 81, Female, N= 86.

^d^Intervention: Least Disadvantaged, N= 48, Most disadvantaged, N=84. Control: Least Disadvantaged, N= 74, Most disadvantaged, N= 86.
